# Long-term survival of advanced lung adenocarcinoma by maintenance chemotherapy followed by EGFR-TKI: A case report

**DOI:** 10.1097/MD.0000000000024688

**Published:** 2021-02-12

**Authors:** Hui Jun Chen, Ji Min Shen, Ying Ni Lin, Wei Du, Jian Ping Zhou, Qing Yun Li

**Affiliations:** a Department of Respiratory and Critical Care Medicine, Ruijin Hospital, Shanghai Jiao Tong University School of Medicine, Shanghai; b Department of Respiratory Medicine, Affiliated Jinhua Hospital, Zhejiang University School of Medicine, Jinhua, Zhejiang Province, China.

**Keywords:** maintenance chemotherapy, nonsmall cell lung cancer, survival, targeted therapy

## Abstract

**Rationale:**

The incidence of nonsmall cell lung cancer (NSCLC) is high. Most nonsmall cell lung cancers have undergone multiple metastases at the time of initial diagnosis, and the 5 year survival rate is low. At present, comprehensive treatments, including systemic chemotherapy, targeted therapy, antiangiogenic therapy, and immunotherapy, prolong the survival of patients with advanced NSCLC. Herein, we report a case of NSCLC with long-term survival.

**Patient concerns:**

A 61-year-old woman complained of dry cough and shortness of breath and visited our hospital in July 2011. Imaging examination revealed a left upper lung mass with multiple metastases to the liver, adrenal gland, and bone.

**Diagnoses:**

Stage IVB (cT2aN3M1c) lung adenocarcinoma was diagnosed, with multiple metastases of the lymph nodes, liver, adrenal gland, and bone.

**Interventions and outcomes:**

The patient received systemic chemotherapy and epidermal growth factor receptor-tyrosine kinase inhibitor-targeted therapy, and has survived for more than 9 years.

**Lessons:**

The patient benefited from maintenance chemotherapy and epidermal growth factor receptor-tyrosine kinase inhibitor treatment and achieved long-term survival.

## 1 Introduction

Nearly 75% of patients with nonsmall cell lung cancer (NSCLC) are diagnosed at an advanced stage.^[1]^ The 5-year survival rate of NSCLC is approximately 15.9%, while that of advanced NSCLC is much lower, ranging from 2.8% to 14.6% .^[2–4]^ During the past decade, many pharmacological developments, including targeted therapy and immunotherapy, have led to the long-term survival of patients with advanced NSCLC. Herein, we report a patient who was diagnosed with NSCLC at stage IVB, benefited from maintenance chemotherapy and subsequent epidermal growth factor receptor-tyrosine kinase inhibitor (EGFR-TKI) treatment after progression of chemotherapy, and has survived for more than 9 years.

## 2 Case report

**Figure 1:**
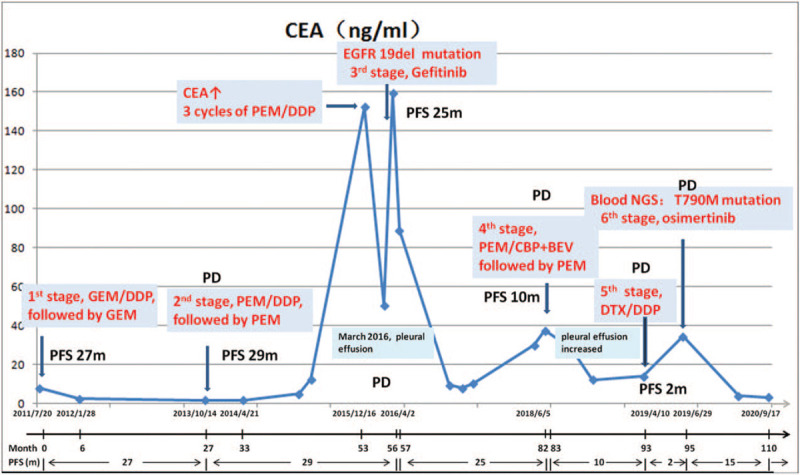
The time line of treatments and the trends of CEA. The serum CEA decreased from 7.96 ng/ml to 1.84 ng/ml after 4 cycles of GEM/DDP treatment. It increased progressively before the 2nd PD, and decreased from 159.42 ng/ml to 16.59 ng/ml half a year after gefitinib treatment. Before the 3rd, 4th and 5th PD, the reaction of CEA was performed prior to imaging findings. When the treatments were effective, the CEA levels decreased significantly. CEA, carcinoembryonic antigen; PD, progressive disease; GEM, gemcitabine; DDP, cisplatin; PEM, pemetrexed; CBP, carboplatin; BEV, bevacizumab; DTX, docetaxel; EGFR, epidermal growth factor receptor; NGS, next-generation sequencing; PFS, progression-free survival.

**Figure 2:**
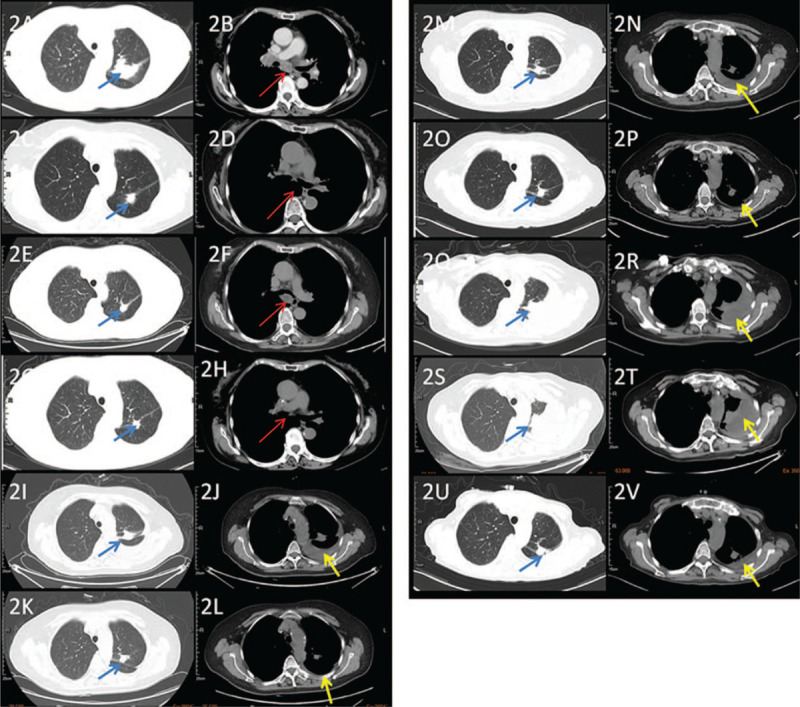
Computed tomography scans showing clinical response to treatments. The patient achieved partial remission after GEM/DDP (A–D). Progressive disease with enlarged subcarinal lymph nodes (F). The subcarinal lymph node disappeared 4 months after treatment with PEM/DDP (H). Lung lesions increased, and malignant pleural effusion developed in March 2016 (I and J). One month after gefitinib treatment, the patient achieved SD with shrinkage of the lung lesions and pleural effusion (K and L). After 25-month PFS, the volume of pleural effusion increased and a new lesion was found near the primary lesion (M and N), which disappeared after 2 cycles of PEM/CBP+BEV (O and P). The disease progressed with enlarged lesions and increased pleural effusion volume (Q and R). There was no benefit from the 2 cycles of DTX/DDP (S and T). Pleural effusion was reduced after osimertinib treatment (V). Blue arrows indicate the lesion, red arrows indicate the subcarinal lymph node, and yellow arrows indicate pleural effusion. GEM, gemcitabine; DDP, cisplatin; SD, stable disease; PEM, pemetrexed; CBP, carboplatin; BEV, bevacizumab; DTX, docetaxel.

A 61-year-old woman, with a history of secondhand smoke exposure and family history of lung cancer, complained of dry cough and shortness of breath, and visited our hospital in July 2011. Chest enhanced computed tomography (CT) showed lung cancer in the dorsal segment of the left upper lobe, accompanied by metastasis of the cervical root, subclavian fossa, bilateral hilar, mediastinal lymph nodes, and liver. An enhanced CT scan of the upper abdomen showed multiple intrahepatic and left adrenal metastases. Superficial lymph node B ultrasound showed bilateral neck, supraclavicular, and axillary lymph node metastases. Bone single photon emission CT with CT revealed multiple punctate lesions in the spine. Magnetic resonance imaging of the thoracic and lumbar vertebrae showed multiple metastases of the thoracic and lumbar vertebrae. No significant intracranial metastasis was observed on enhanced magnetic resonance imaging. CT-guided biopsy of the left upper lung mass led to a diagnosis of lung adenocarcinoma. The EGFR driving gene was negatively detected by real-time PCR. Stage IVB (cT2aN3M1c) lung adenocarcinoma was diagnosed according to the TNM classification of the Union of International Cancer Control 8th edition, with multiple metastases to the liver, adrenal gland, and bone. The Eastern Cooperative Oncology Group performance status score was 1. Figure 1 shows the timeline of treatments and trends of carcinoembryonic antigen (CEA). The patient received 6 cycles of first-line chemotherapy with gemcitabine/cisplatin (DDP) from July 2011 to November 2011. Partial remission (PR) was achieved with a decrease in tumor size and disappearance of the subcarinal lymph node, according to the Response Evaluation Criteria in Solid Tumors version 1.1 (Fig. 2A-2D). The patient then underwent 14 cycles of gemcitabine maintenance therapy. The liver metastases disappeared after 9 months. In October 2013, the patient presented with progressive enlargement of the subcarinal lymph node after 27-month progression-free survival (PFS) (Fig. 2E and F), and received 6 cycles of second-line chemotherapy with pemetrexed (PEM)/DDP followed by 18 cycles of PEM maintenance. The patient achieved PR (Fig. 2G and H) and a second long-term PFS of 29 months. In December 2015, the patient experienced a rebound of serum CEA levels and received another 3 cycles of PEM/DDP. In March 2016, the patient developed malignant pleural effusion (PE) (Fig. 2I and J). Genetic analysis of circulating tumor DNA in PE demonstrated an EGFR 19-del mutation. Consequently, the patient has been prescribed gefitinib (250 mg daily) since April 2016, and PE decreased after a month (Fig. 2K and L). In June 2018, after 25-month of PFS, the patient presented with a new lesion near the primary lesion and an increased PE volume (Fig. 2M and N). The patient could not undergo gene tests and osimertinib. Thus, PEM/carboplatin combined with bevacizumab was administered for 6 cycles, followed by 4 cycles of PEM maintenance therapy, and the condition was controlled (Fig. 2O and P). The disease did not progress until April 2019 (Fig. 2Q and R), and the patient did not respond to 2 cycles of docetaxel/DDP (Fig. 2S and T). Genetic analysis of circulating tumor DNA in blood revealed EGFR T790M mutation (abundance 2%) and EGFR 19-del mutation (abundance 1%). Osimertinib (80 mg daily) was administered in June 2019 when osimertinib was covered by insurance. At present, the patient has achieved PR (Fig. 2U and V) and 15-month of PFS, and was still alive at the most recent follow-up, with mild headache and oral ulcer, and could perform general housework.

Written informed consent was obtained from the patient for publication of this report.

## 3 Discussion

Herein, we report the case of a patient who had survived for more than 9 years. The patient was diagnosed with advanced NSCLC with multiple extra-thoracic metastases and negative EGFR mutations, and benefited from chemotherapy followed by EGFR-TKI. Several aspects must be considered. First, the patient had an initial good PS and did not develop brain metastasis, which are predictors of long-term survival in advanced NSCLC.^[3,4]^ Second, significant reductions in serum CEA levels after initiating treatment may predict longer overall survival.^[5]^ In our case, the serum CEA level changed significantly with progression and reflected the response to therapy. Third, maintenance chemotherapy significantly improved PFS and overall survival and reduced toxicity.^[6–9]^ A patient with good PS tolerated repeated cycles of platinum-based doublet therapy and continuation maintenance chemotherapy, and achieved long-term PFS. Fourth, chemotherapy may influence EGFR mutation status.^[10]^ The patient was initially negative for EGFR mutations, but tested positive for EGFR 19-del mutation after she declined chemotherapy and achieved a 25-month long PFS with first-generation EGFR-TKI. The patient acquired EGFR resistance mutation T790M after the failure of gefitinib and responded well to the third-generation EGFR-TKI osimertinib. Finally, patients with advanced NSCLC are always concerned about the cost of anticancer therapy, which demands the improvement of healthcare system.^[11]^ For those who have good PS and cannot undergo gene tests and targeted therapy, platinum-based doublet therapy followed by maintenance chemotherapy could be the choice.

## 4 Conclusion

Here, we report a case of stage IVB lung adenocarcinoma without EGFR mutation at initial diagnosis. The patient achieved first- and second-line platinum-based doublet therapy and achieved a long PFS by continuation of maintenance chemotherapy. After progression to second-line chemotherapy, the patient tested positive for EGFR mutations, and was administered EGFR-TKI. After 25-month PFS with gefitinib, the patient refused gene tests until osimertinib was covered by medical insurance. The patient has benefited from maintenance chemotherapy and EGFR-TKI treatment and has survived for more than 9 years.

## Author contributions

**Conceptualization:** Hui Jun Chen, Qing Yun Li.

**Data curation:** Hui Jun Chen, Ji Min Shen, Ying Ni Lin.

**Supervision:** Qing Yun Li.

**Writing – original draft:** Hui Jun Chen, Ji Min Shen, Ying Ni Lin.

**Writing – review & editing:** Wei Du, Jian Ping Zhou, Qing Yun Li.

## Abbreviations

Abbreviations: CEA = carcinoembryonic antigen, CT = computed tomography, DDP = cisplatin, DTX = docetaxel, EGFR = epidermal growth factor receptor, EGFR-TKI = epidermal growth factor receptor-tyrosine kinase inhibitor, MRI = magnetic resonance imaging, NSCLC = non-small cell lung cancer, PEM = pemetrexed, PFS = progression-free survival, PR = partial remission.
